# The Role of Lactate Exercise Test and Fasting Plasma C-Peptide Levels in the Diagnosis of Mitochondrial Diabetes: Analysis of Clinical Characteristics of 12 Patients With Mitochondrial Diabetes in a Single Center With Long-Term Follow-Up

**DOI:** 10.3389/fendo.2022.835570

**Published:** 2022-02-21

**Authors:** Yuan Zhao, Ying Zhang, Mengya Qi, Fan Ping, Huabing Zhang, Lingling Xu, Wei Li, Yuxiu Li

**Affiliations:** ^1^ Key Laboratory of Endocrinology of National Health Commission, Department of Endocrinology, Peking Union Medical College Hospital, Chinese Academy of Medical Sciences & Peking Union Medical College, Beijing, China; ^2^ Department of Endocrinology, The Hospital of Shunyi District, Beijing, China

**Keywords:** mitochondrial diabetes, clinical features, fasting C-peptide, lactate exercise test, monogenic diabetes

## Abstract

**Objective:**

The aim of this study was to analyze the clinical characteristics and the pattern of long-term changes of fasting plasma C-peptide in patients with mitochondrial diabetes (MD), and to provide guidance for the diagnosis and treatment of MD.

**Methods:**

We retrieved MD patients with long-term follow-up at Peking Union Medical College Hospital from January 2005 to July 2021 through the medical record retrieval system and retrospectively analyzed their clinical data, biochemical parameters, fasting plasma C-peptide, glycosylated hemoglobin and treatment regimens. Non-parametric receiver operating characteristic (ROC) curves were used to assess the relationship between exercise test-related plasma lactate levels and suffering from MD.

**Results:**

A total of 12 MD patients were included, with clinical characteristics of early-onset, normal or low body weight, hearing loss, maternal inheritance, and negative islet-related autoantibodies. In addition, patients with MD exhibited significantly higher mean plasma lactate levels immediately after exercise compared to patients with type 1 diabetes mellitus (T1DM) (8.39 ± 2.75 vs. 3.53 ± 1.47 mmol/L, p=0.003) and delayed recovery time after exercise (6.02 ± 2.65 vs. 2.17 ± 1.27 mmol/L, p=0.011). The optimal cut-off points identified were 5.5 and 3.4 mmol/L for plasma lactate levels immediately after and 30 minutes after exercise, respectively. The fasting plasma C-peptide levels decreased as a negative exponential function with disease progression (Y= 1.343*e^-0.07776X^, R^2^ = 0.4154). Treatment regimens in MD patients were varied, with no metformin users and a weak correlation between insulin dosage and body weight.

**Conclusions:**

The increased level of plasma lactate during exercise or its delayed recovery after exercise would contribute to the diagnosis of MD. Changes of fasting plasma C-peptide in MD patients over the course of the disease indicated a rapid decline in the early stages, followed by a gradual slowing rate of decline.

## Introduction

Mitochondrial diabetes (MD) is a rare monogenic form of diabetes caused by defects in mitochondrial DNA (MtDNA) function (1), accounting for only 0.6% of adults with diabetes in China (2). van den Ouweland JM et al. (3) and Ballinger et al. (4) first reported a family of MD with elevated blood glucose and deafness caused by mutations and large segmental deletions of mitochondrial gene in 1992. Mitochondrial gene mutations are highly heterogeneous and individuals with the same mutation often exhibit different types and degrees of clinical phenotype, resulting in MD being diagnosed as type 1 or type 2 diabetes mellitus frequently.

The main pathogenesis of MD is mitochondrial gene mutation which leads to the decrease of insulin secretion by islet βcells (5). At present, the changes of islet βcell function in patients with MD have only been reported in individual cases or small families, and there is still a lack of large and long-term follow-up studies. In this study, we retrospectively analyzed and summarized the MD patients followed up in the outpatient clinic of Peking Union Medical College Hospital, in order to provide reference for understanding the changes of islet βcell function and the diagnosis and treatment of MD.

## Patients and Methods

### Population

A total of 49 patients diagnosed with MD in the outpatient clinic of Peking Union Medical College Hospital from January 2005 to July 2021 were searched through the electronic medical record system. Further review of the medical records revealed 15 cases with clear mitochondrial gene mutations. To analyze the changes in pancreatic β-cell function during follow-up in these patients, the inclusion criteria were set as follows: follow-up for at least one year, and fasting plasma C-peptide test twice or more. A total of 12 patients met the criteria, and the longest follow-up period was 16 years, and a retrospective analysis of these 12 patients with MD was performed.

### Data Collection

General information of patients was collected: age, sex, height, weight, time of onset, hearing status, family history. The results of islet-related autoantibodies including glutamic acid decarboxylase antibody (GADAb), protein tyrosine phosphatase antibody (IA2) and islet cell antibody (ICA), mitochondrial gene sequencing, blood biochemical parameters, plasma lactate, HbA1c, fasting plasma C-peptide and therapeutic regimen (types and doses of hypoglycemic drugs) at each visit were recorded.

Six MD patients had undergone lactate exercise testing, thus we included six patients with type 1 diabetes mellitus (T1DM) who had performed the same test as controls for comparison by matched search in the medical record retrieval system according to age and HbA1c at the time of the test.

Method of lactate exercise test: After a 30-minute rest period before the test, venous blood was taken for lactate measurement, followed by 10 minutes of stair climbing at moderate speed without stopping, and venous blood was taken immediately afterward, and the third blood sample after a 30-minute rest. The plasma lactate level was measured separately and >2 mmol/L was considered abnormal.

### Statistical Analysis

Statistical analyses were performed using IBM SPSS Statistics for Windows, version 26 (IBMCorp., Armonk, N.Y., USA). Continuous data were expressed as mean ± standard deviation when normally distributed, and t-test was used for comparison of data between groups, and P values ≤ 0.05 were considered to be statistically significant. Non-parametric receiver operating characteristic (ROC) curves were constructed for plasma lactate levels immediately after and 30 minutes after exercise to assess their ability to independently discriminate between patients with and without MD. Optimal cut-off points were obtained by minimizing the distance between each point on the ROC curve and the upper left corner.

## Results

### Analysis of Clinical Characteristics of MD Patients

Among the 12 patients with MD, the age of onset was 30.17 ± 10.57 years, 11 (92.3%) of them were younger than 45 years (**Table 1**). Their weight was normal or low with a mean BMI of 18.61 ± 4.14 kg/m2 and only one case was overweight (27.36 kg/m2) and no obese patients. Eleven cases had hearing loss. The health status of the maternal and her family was unknown in one case, and the remaining 11 cases all had a family history of maternal hereditary diabetes or hearing loss. All patients were negative for GADAb, IA2, and ICA. The details of mitochondrial gene mutations were as follows: 9 cases with *m.3243 A>G* mutation, 1 case with multi-point mutations (*m.3243 A>G, m.1438 A>G, m.3206 C>T, m.14783 T>C, m.16213 C>T, m.16319 G>A*), 1 case with *m.15662 A>G* mutation and 1 case with *m.1438 A>G* mutation.

**Table 1:** Demographic and clinical characteristics of 12 patients with MD.

Patient	Diagnosis	Gender	Age at onset, y	Age at Baseline, y	Follow-Up time, y	Height, cm	Weight, kg	BMI (kg/m^2^)	Resting lactate, mmol/L	Family history of	
										Diabetes	Hearing abnormalities
1	*3243 A>G*	F	38	41	14	157	39.5	16.02	–	Mother, sister, daughter	Mother, daughter, sister, niece
2	*3243 A>G*	F	15	18	14	157	54	21.91	1.67	Mother, grandmother, aunt	Mother, grandmother, aunt, aunt’s daughter
3	*3243 A>G*	M	36	48	9	170	57	19.72	1.7-1.9	Mother	–
4	Mult mtDNA PM	F	11	11	16	159	57	22.55	1.7-3.1	Father, mother, uncle, grandfather, grandmother	–
5	*3243 A>G*	M	37	51	3	168	58	20.55	1.9	Aunt, brothers	Brothers
6	*m.15662 A>G*	M	23	24	3	171	80	27.36	0.76-2.7	–	–
7	*3243 A>G*	F	40	43	2	152	33	14.28	2.3-2.7	–	Sister, daughter
8	*3243 A>G*	F	37	38	4	150	45	20	1.8	Brother	Mother, brother
9	*3243 A>G*	F	27	41	3	159	39	15.43	2.6	Mother, brother, aunt	Mother, brother, uncles
10	*3243 A>G*	F	26	29	2	160	42	16.41	1.5	Father, mother, grandfather, grandmother, uncle	–
11	*m.1438 A>G*	M	26	26	1	172	47.5	16.06	0.98	Father, aunt, uncle	Sister
12	*3243 A>G*	F	46	50	6	157	32	12.98	–	Mother, sister, Niece	Mother, daughter, sister, niece

BMI, body mass index; M/F, male/female; PM, point mutation.

### The Level of Plasma Lactate Increased Significantly During and After Exercise in Patients With MD

In 10 of the 12 patients with MD, plasma lactate was measured at rest and its measurements ranged from 0.76 to 3.3 mmol/L (**Table 1**), with fluctuations not significantly related to the duration of the disease. P Plasma lactate at rest was detected only once in six patients, five of whom were within the normal range (0.98-1.9 mmol/L), and one was elevated (2.6 mmol/L). Four patients were tested several times for plasma lactate at rest: P#3 was tested twice, and the results were normal (1.7-1.9 mmol/L); P#7 was tested for three times, and the results were all increased (2.3-2.7 mmol/L); P#4 was tested for five times, and the results were normal (1.7-1.79 mmol/L) in two times and increased (2.31-3.3 mmol/L) in three times; P#6 was tested for three times, and the results were normal (0.76 mmol/L) in one time and increased (2.05-2.7 mmol/L) in two times.

**Figure 1:**
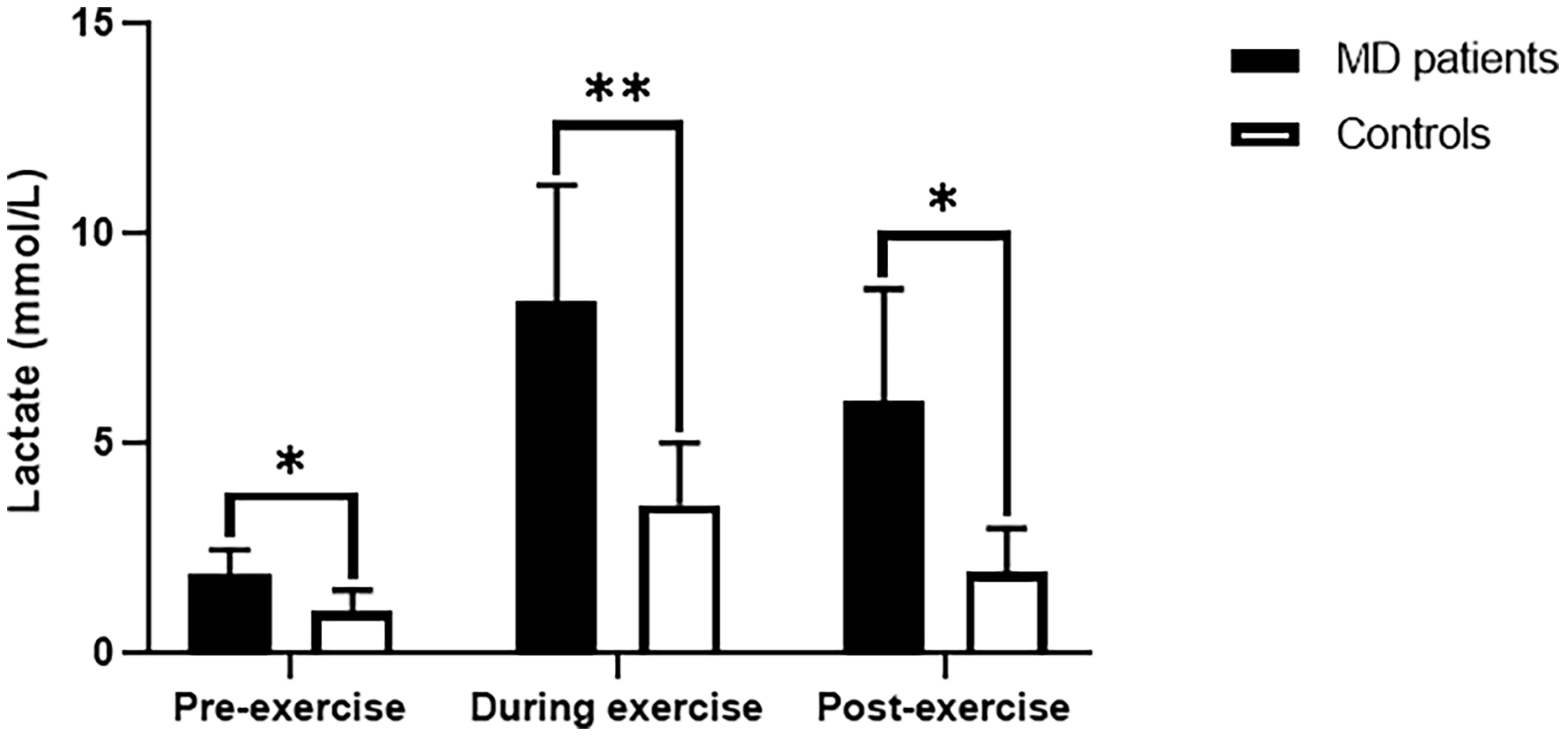
Lactate values for exercise tests in MD patients and T1DM patients. MD, Mitochondrial diabetes; T1DM, Type 1 diabetes mellitus. *p < 0.05,**p < 0.01.

Six MD patients and six T1DM patients underwent lactate exercise testing, with plasma lactate levels measured before, immediately after, and 30 minutes after exercise (**Table 2**). As shown in **Figure 1**, lactate was significantly higher in MD patients immediately after exercise compared to resting lactate and was 2-3fold higher than in T1DM patients (8.39 ± 2.75 vs. 3.53 ± 1.47 mmol/L, p=0.003). Thirty minutes after the exercise, lactate did not drop to normal levels in MD patients and was 3-4fold higher than in T1DM patients (6.02 ± 2.65 vs. 2.17 ± 1.27 mmol/L, p=0.011). Using nonparametric ROC curve, for plasma lactate levels immediately after exercise (area under the curve (AUC) 0.94, 95% confidence interval (c.i.) 0.81 to 1), the optimal cut-off was 5.5 mmol/L with a sensitivity of 83% and specificity of 100%. For plasma lactate levels 30 minutes after exercise (AUC 0.96, 95% c.i. 0.85 to 1), the optimal cut-off was 3.4 mmol/L, giving 100% sensitivity and 83% specificity (**Supplementary Figure 1**).

**Table 2:** Demographic characteristics of MD patients and T1DM patients.

	MD patients (n = 6)	T1DM patients^#^ (n = 6)	p-value
Gender, male, n (%)	3 (66.7%)	1 (16.7%)	–
Age at onset, y	30.17 ± 10.57	40.5 ± 14.85	0.242
Age at exercise tests, y	42.58 ± 12.06	46.75 ± 16.72	0.593
Height, cm	161 ± 7.45	166.17 ± 5.6	0.155
Weight, kg	48.67 ± 13.47	64.23 ± 8.25	0.02
BMI, kg/m^2^	18.61 ± 4.14	23.29 ± 2.98	0.026
Fasting C-peptide, ng/ml	0.59 ± 0.31	0.09 ± 0.02	0.007
HbA1c, %	7.93 ± 1.49	6.9 ± 1.02	0.148

MD, Mitochondrial diabetes; T1DM, Type 1 diabetes mellitus. ^#^Clinical and laboratory findings of patients with T1DM were obtained from our institution’s medical record retrieval system matched by age and HbA1c at the time of the test.

### Changes of Fasting Plasma C-Peptide Level With Disease Duration

**Figure 2:**
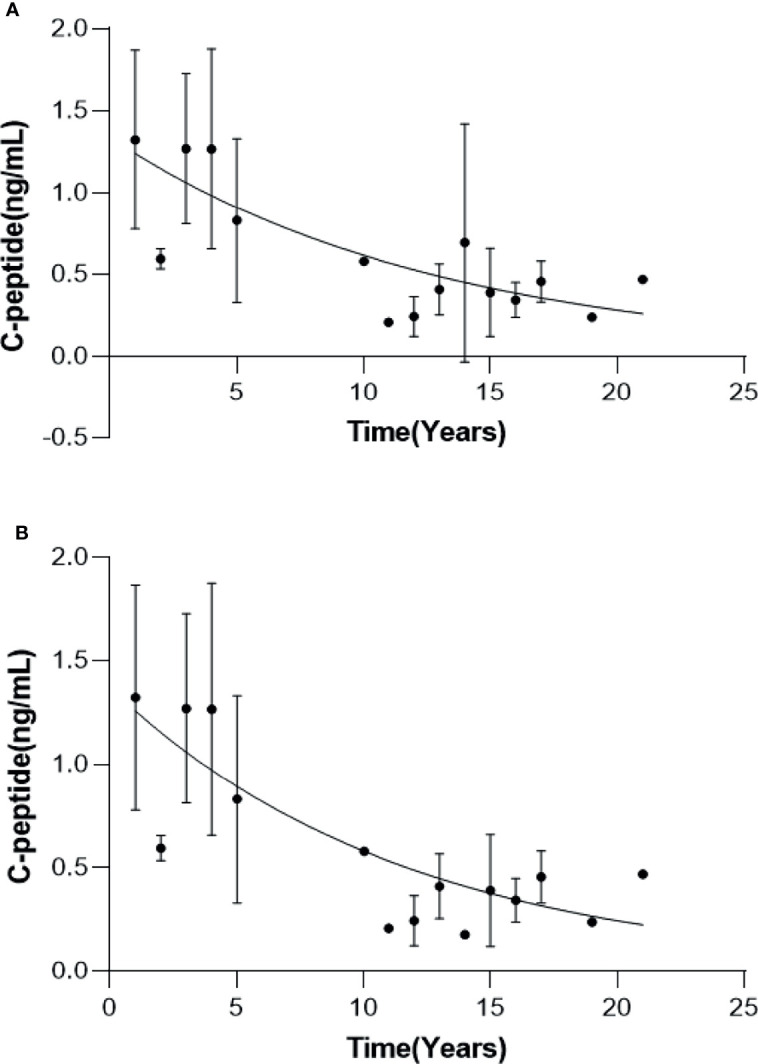
Changes in fasting C-peptide with the duration of disease in MD patients. Curves of fasting C-peptide with disease duration for all MD patients **(A)** and MD patients except P#4 **(B)**.

All 12 MD patients were tested for fasting plasma C-peptide at a median of three times (range 2-7 times) over a median of 13 years (range 2-21 years) after onset, and their C-peptide levels varied with the disease duration (**Figure 2A**). The fasting plasma C-peptide was 0.7-1.72 ng/ml with an average of 1.92 ng/ml in the first year and was 0.31-1.31 ng/ml with an average of 0.83 ng/ml in the fifth year. The fitting exponential curve of the data indicated that fasting plasma C-peptide decreased rapidly with the duration of the disease in the first 5 years (Y= 1.343*e^-0.07776X^, R^2^ = 0.4154). After 10 years of disease duration, the fasting plasma C-peptide level was maintained at a low level (0.16-0.79ng/ml) with no significant trend of further decline, except for P#9 which reached 1.21 ng/mL by the 14th year of disease duration. The fitting exponential curve reanalyzed deleting this value of P#9 showed that the decrease in fasting plasma C-peptide remained a negative exponential function with a steeper curve than in **Figure 2A** (Y= 1.371*e^-0.08597X^, R^2^ = 0.4652) (**Figure 2B**).

### Comparison of Treatment Regimens Between the First and the Last Visit of 12 Patients With MD

Among the 12 patients, HbA1c of 10 patients fluctuated between 6.2 and 8.6% in 10 patients (**Table 3**), that of another 2 patients were above 10%, of which P#4 did not follow medical advice on diet control and insulin use, and P#11 only got a definite diagnosis of MD at the last visit and adjusted the glucose-lowering regimen but was not followed up later. Metformin was included in the treatment regimen of three patients at the first visit and before the diagnosis of MD was clarified. After the diagnosis of MD was confirmed and at the last follow-up visit, 11 patients used insulin, including one case combined with glucagon-like receptor agonist (GLP-1RA) and three cases combined with sodium-glucose co-transport protein 2 inhibitor (SGLT-2i), without adverse effects such as malaise, ketosis or ketoacidosis, or severe gastrointestinal reactions. One case used oral hypoglycemic agents only (glimepiride 1 mg once daily, HbA1c 6.2%). Metformin was not used. Except for P#4 and P#11, HbA1c decreased significantly at the last visit compared with the first visit.

**Table 3:** HbA1c values and treatment regimens before and after follow-up.

Patient	First Visit			Last Visit			
	Disease Duration (y)	HbA1c (%)	Treatment	Disease Duration (y)	HbA1c (%)	Treatment	
						Insulin (Dose U/kg/d)	Other Antidiabetic Drugs (Dose)
1	3	13	Premixed human insulin 30R	17	7.4	0.35 (Premixed insulin aspart 50)	–
2	3	7.8	Intensive insulin therapy	17	8.6	1.19 (Aspart, detemir)	–
3	13	11.6	Oral drugs and insulin	21	8	0.91 (Aspart, glargine)	Linagliptin (5mg qd), dapagliflozin (10mg qd)
4	1	–	Premixed human insulin 30R	16	10.9	1.05 (Glulisine, degludec)	Sitagliptin (100mg qd), canagliflozin (100mg qd)
5	14	9.1	Metformin, vildagliptin	17	7.2	0.43 (Glulisine, degludec)	Acarbose (50mg tid), sitagliptin (100mg qd)
6	2	5.9	Metformin, glimepiride	4	7.2	0.38 (Aspart, degludec)	Dulaglutide (1.5mg qw), dapagliflozin (10mg qd)
7	3	7.9	Insulin glargine	5	6.4	0.48 (Glulisine, glargine)	–
8	1	6.3	Insulin	5	7.2	0.09 (Degludec)	Voglibose (0.3mg tid), sitagliptin (100mg qd)
9	14	9.3	Acarbose	17	8.6	1.23 (Aspart, glargine)	Pioglitazone (15mg qd), acarbose (100mg tid), sitagliptin (100mg qd)
10	3	9.3	Metformin	5	6.2	–	Glimepiride (1mg qd)
11	0	12.9	Insulin aspart, insulin glargine	1	10.5	0.53 (Premixed insulin aspart 30)	–
12	4	7.5	Acarbose	11	7	0.47 (Premixed insulin lispro 50)	–

HbA1c, hemoglobin A1c.

## Discussion

MD is very rare, with individual cases or small families reported in China and no large long-term follow-up studies. In this study, we retrospectively analyzed the clinical data of 12 patients with MD with a maximum follow-up of 16 years and concluded that MD has the following clinical features: early age of onset, thin or normal body size, family history of maternal-inherited diabetes or hearing loss, and negative for islet-related antibodies (ICA, GAD, IA2), which is consistent with the literature (6). Patients with these characteristics need to be considered for possible MD. The diagnosis of MD currently relies on mitochondrial gene sequencing, but few hospitals are able to perform this test.

In the analysis of the clinical data of the 12 patients followed in this study, it was found that the lactate exercise test was a good indication for diagnosis, the large variation in blood lactate at rest was not significant for diagnosis. There are only a few reports on blood lactate in MD cases or families at rest (7–10), with inconsistent results, some elevated and some normal, with high variability, and no reports on lactate changes during and after exercise. Mitochondria are organelles that carry out aerobic metabolism and oxidative phosphorylation production and are responsible for 90% or more of the body’s ATP supply. Mutations in MtDNA result in oxidative phosphorylation dysfunction and reduced ATP production. This is followed by an increase in anaerobic enzyme production capacity and an increase in lactate production (11). At rest, the magnitude of blood lactate elevation depends on the degree of disease heterogeneity (12). During exercise, patients have elevated blood lactate at any given work rate compared to normal controls (13). There are no differences in serum lactate levels at rest, during and after exercise with and without clinical signs of myopathy (14). Of the 12 MD patients followed in this study, 10 patients underwent a total of 19 resting-state blood lactate tests, 10 had normal test results (0.76-1.9 mmol/L) and 9 had elevated test results (2.05-3.3 mmol/L). There was significant variability in the results of multiple measurements in the same patient with inconsistent results. This suggests that blood lactate at rest is highly variable and has limited value as a diagnostic screening indicator for patients with MD. The lactate exercise test was performed on six patients in this study, and lactate was significantly elevated during exercise, none of the blood lactate returned to resting levels after exercise. The ROC curves indicated that blood lactate during and after exercise had high diagnostic efficacy with a specificity and sensitivity of 100%, respectively, and the optimal cut-off points identified were 5.5 and 3.4 mmol/L, respectively. The lactate exercise test may be a useful screening method for MD when genetic testing is not available. To our knowledge, this is the first study to analyze the characteristics of blood lactate after exercise in MD patients and to assess its diagnostic ability. Thus, the accuracy of this test was not compared with other diagnostic tests, and further studies with larger sample sizes are needed to validate these findings.

Because of the low and rare incidence of MD, changes in islet cell function in MD have only been reported in individual cases or small family lines with short-term follow-up, and there are no large long-term follow-up studies. In this study, the first retrospective analysis of fasting C-peptide levels in 12 MD patients over a maximum of 16 years revealed that fasting C-peptide in MD patients decreased as a negative exponential function over the course of the disease. The decline in fasting C-peptide was faster early in the course of the disease, slowed down as the disease progressed, and remained in small amounts for more than 10 years of the disease. This is distinctly different from type 1 diabetes, which undergoes complete islet cell failure in a very short period of time. Yoshitomo Oka et al. (15) reported a similar case with similar follow-up (rapid decline in fasting C-peptide over 5 years, maintained at low levels after 11 years). The characteristics of the fasting C-peptide decline are related to the pathogenesis of MD. Mitochondria are responsible for a variety of biological functions, the most critical of which is the production of ATP and reactive oxygen species (ROS), the latter of which can cause cellular damage. Mutations in mitochondrial genes cause cellular alterations such as decreased ATP production, increased ROS, lipid peroxidation, altered membrane potential and increased apoptotic signaling, which in turn can lead to cellular damage and apoptosis. When the mutation is located in the islet, slow destruction of β-cells may occur, leading to decreased insulin secretion and the development of MD (11). MD is a highly heterogeneous disease in which each β-cell contains both mutant and wild-type mtDNA, referred to as heterogeneity, and different degrees of mutant mtDNA exist in different β-cells of the same individual (12). The ratio of mutant to wild-type mtDNA is a key factor in determining whether β-cells express biochemical abnormalities (16). Islet cells containing more mutant mtDNA develop dysfunction earlier and faster, leading to premature apoptosis (17). Our previous study also showed that individuals with high amounts of mutations had earlier onset and more severe disease (18). Islet cells containing less mutated mtDNA with wild-type mtDNA still maintain normal cellular function and can avoid initiating the apoptotic program and maintain normal β-cell function in the long term. For diabetic patients with lean body type, poor islet cell function and negative diabetes-related antibodies, and still detectable islet cell function after a longer disease course, we should be alert to the possibility of MD.

There is no evidence-based medical evidence to guide the treatment of MD. Early initiation of insulin therapy is recommended because of the rapid decline in islet cell function during the early stages of the disease. Metformin should be avoided in the treatment of MD because of the risk of lactic acidosis. Our follow-up MD patients used a variety of hypoglycemic regimens, including one case with GLP-1RA and three cases with SGLT-2i, with no significant adverse effects and all with a significant decrease in glycated hemoglobin, similar to the treatment effects reported in the literature (19, 20). It indicates that the treatment of MD patients can be diversified according to the specifics of blood glucose and islet cell function, and that the novel hypoglycemic drugs are effective without significant adverse effects. However, due to the small number of cases we observed and the limited follow-up time, whether these novel hypoglycemic drugs are safe for MD patients still needs to be observed in long-term follow-up.

In conclusion, we should consider the possibility of MD in patients with young to middle-aged onset, thin or normal body size, hearing loss, family history of maternal-inherited diabetes or hearing loss, negative antibodies (IAA, ICA, GAD), and residual islet cell function after a long course of disease. The lactate exercise test can be further completed. If there is an increase in plasma lactate level during exercise and delayed recovery after exercise, it is necessary to be highly alert to the possibility of MD. The diagnosis can be confirmed by hot spot detection of mitochondrial gene mutation. The treatment of MD should be individualized according to the specific conditions such as blood glucose and islet cell function, as well as avoidance of metformin. However, our sample is relatively small, and a larger population-based cohort and more longitudinal studies are warranted.

## Data Availability Statement

The raw data supporting the conclusions of this article will be made available by the authors, without undue reservation.

## Ethics Statement

The studies involving human participants were reviewed and approved by Ethics Committee of the Peking Union Medical College Hospital. Written informed consent for participation was not required for this study in accordance with the national legislation and the institutional requirements.

## Author Contributions

YAZ and YZ: data acquisition and drafting of the manuscript. MQ, FP, HZ, and LX: data acquisition and analysis and interpretation of the data. WL and YL: study concept and design, critical revision of the manuscript for important intellectual content, and study supervision. All authors contributed to the article and approved the submitted version.

## Funding

This research is supported by the CAMS Innovation Fund for Medical Sciences (CIFMS)2021-I2M-1-002.

## Conflict of Interest

The authors declare that the research was conducted in the absence of any commercial or financial relationships that could be construed as a potential conflict of interest.

## Publisher’s Note

All claims expressed in this article are solely those of the authors and do not necessarily represent those of their affiliated organizations, or those of the publisher, the editors and the reviewers. Any product that may be evaluated in this article, or claim that may be made by its manufacturer, is not guaranteed or endorsed by the publisher.
