# Supplementary figures and images for: The Role of Lactate Exercise Test and Fasting Plasma C-Peptide Levels in the Diagnosis of Mitochondrial Diabetes: Analysis of Clinical Characteristics of 12 Patients With Mitochondrial Diabetes in a Single Center With Long-Term Follow-Up
Source: Front Endocrinol (Lausanne). 2022 Feb 21;13:835570. doi: 10.3389/fendo.2022.835570 (PMC8899008; doi:10.3389/fendo.2022.835570)

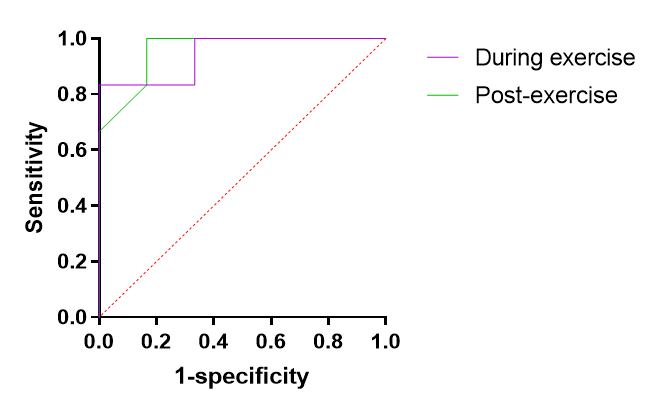

Supplement: Supplementary file 1 [file Image_1.tif]
